# Vulnerability to Suicide Ideation: Comparative Study between Adolescents with and without Psychosocial Risk

**DOI:** 10.3390/healthcare11192663

**Published:** 2023-10-01

**Authors:** Marta Brás, Patrícia Elias, Francisca Ferreira Cunha, Cátia Martins, Cristina Nunes, Cláudia Carmo

**Affiliations:** 1 Psychology Research Centre (CIP), Universidade do Algarve, Campus de Gambelas, 8005-139 Faro, Portugal; fmcunha@ualg.pt (F.F.C.); csmartins@ualg.pt (C.M.); csnunes@ualg.pt (C.N.); cgcarmo@ualg.pt (C.C.); 2 Universidade do Algarve, Campus de Gambelas, 8005-139 Faro, Portugal; eliaspatricia@aoa.pt

**Keywords:** adolescents, suicidal ideation, mental health, psychosocial risk, social support

## Abstract

Adolescents are characterized as a risk group for suicide, being the fourth leading cause of death in young people. The main aim was to compare vulnerability to suicidal ideation in a sample of young people with and without psychosocial risk. The total sample consisted of 137 adolescents, aged between 10 and 19 years (*M* = 14.76; *SD* = 1.40), and it was composed of two groups—the psychosocial risk group (*n* = 60) and general population group (*n* = 77). In both groups, suicidal ideation correlated positively with negative events and negatively with self-esteem and social support satisfaction. When comparing the two groups, the psychosocial risk group presented significantly higher mean values of negative life events (mainly separations/losses and physical and sexual abuse) and significantly lower mean values of satisfaction with social support (particularly with family and social activities). It was also found that, in the psychosocial risk group, negative life events were the only significant predictors of suicidal ideation. This study allowed identifying the role of risk and protective factors in suicidal ideation, according to the psychosocial risk of adolescents. The practical implications of the findings on adolescents’ mental health and the promotion of their well-being are discussed.

## 1. Introduction

Suicide is a complex and multifaceted phenomenon, resulting from the interaction of several biopsychosocial factors [1]. The incidence of suicide attempts peaks during the mid-adolescent years, and suicide mortality is the fourth leading cause of death in young people between the ages of 10 and 24 [2]. In Portugal, a mortality rate was of 8.9% per 100,000 inhabitants in 2019, also being one of the main causes of death among young adults [3].

Suicide—death intentionally caused by the individual themselves—is the result of a suicidal process, which usually begins with suicidal ideation and can go on to suicide attempts—an intentional act with a non-fatal outcome. Suicidal ideation consists of a set of thoughts about ending one’s own life. A higher frequency of suicidal ideation and the conception of a plan to carry out the act can be indicators of a higher risk of attempted suicide or suicide. For this reason, assessing the level of suicidal ideation and associated risk factors—often beginning in adolescence—is fundamental for developing prevention strategies [4].

There are distinct differences in the characteristics of suicidality between adolescents and adults [5]. Consequently, there is a pressing need to know the factors that can effectively identify young people who are at higher risk of suicide. While depression is strongly linked to suicidality among adolescents [6], it is not always present in every case [7], highlighting the complex interplay of multiple factors contributing to suicidal behavior. Being able to predict which adolescents are more likely to repeat suicidal behavior would greatly aid in establishing preventive measures and intervention strategies for suicidality in this vulnerable population [8].

Studies on suicidal ideation in adolescents emphasize that there is a positive relationship between family cohesion and support with strong social support, and that in these situations, there is less suicidal ideation [9,10,11]. Research on different forms of family functioning regarding suicidal adolescents describe the family’s lack of ability to adapt to change and scarce problem-solving strategies due to poor intra-family communication, so the adolescent’s suicidal behavior can be related to the perception of family functioning [12].

Negative life events and adversity in the family context are a type of risk factor, which includes parental separation or divorce, death of one of the parents, history of physical or sexual abuse, mental illness in the family, family history of suicidal behaviors, bullying, and alcohol or drug abuse among others. Examples of psychological risk factors include impulsivity, low self-esteem, and hopelessness [13]. Several studies suggest that negative life events are a common denominator for most people who commit suicidal acts and, in addition, predispose the development of certain characteristics, such as hopelessness [14,15].

Hopelessness is an individual risk factor referenced in several models [16,17] that makes people interpret current problems as irresolvable [18]. Also, adverse living conditions together with personal characteristics (e.g., low educational level, scarce or inadequate problem-solving strategies, impulsivity, and lack of self-control) may trigger risky family environments among families at psychosocial risk [19]. This may affect the various family members, particularly the well-being and biopsychosocial development of children and adolescents, due to the chaotic scenario and dysfunctional communication. Studies show that the smaller the displays of affection and the weak configuration of family rules, the greater the psychological stress and the decrease in problem-solving skills [20].

Adolescents from families at psychosocial risk effectively experience several negative life events and seem to have low expectations for the future, thus the contribution of these factors to suicidal ideation seems relevant to study, since suicide in adolescence is a tragic experience at the individual, family, and community levels [21]. In this sense, adolescents with psychosocial risk, caused by family dysfunction, are more vulnerable to suicidal ideation [10,22].

In an opposite direction, there are the protective factors, which reduce the risk of suicide and can be considered “insulators” against suicide, which include family support; friendships and other significant relationships’ support; religious, cultural, and ethnic beliefs; community involvement; satisfactory social life; good social integration; and access to mental health services and care [23]. In terms of individual variables, reasons for living and self-esteem can function as protectors, since when they exist, even in the presence of risk factors, they lead to a lower probability for the suicidal act to occur. The satisfaction with social support, since it is negatively correlated with higher levels of suicidal ideation, also seems to work as a protective factor [18].

Studies suggest that the assessment of the probability of suicidal ideation lies in the balance between risk and protective factors [21], referring to greater or lesser vulnerability. Thus, if young people with psychosocial risk are more vulnerable, they may have higher levels of suicidal ideation than young people who do not present psychosocial risk.

In the Portuguese context, there is a lack of cross-national literature on suicidal ideation in adolescents, especially comparative studies. Studies on suicide risk mainly assess samples from the general population [18] or other specific risk characteristics (e.g., [24]), but to our knowledge, there are few comparative studies [25]. Furthermore, emphasis has been placed on the study of risk factors for suicidal ideation/suicide, to the detriment of studying protective factors [18]. There are also comparative studies that have investigated the mental health of institutionalized versus non-institutionalized adolescents, but they have not assessed the risk of suicide [26]. With this study, we aim to fill the gaps identified and contribute to the advancement of knowledge so that it can be translated into suicide prevention strategies.

The main aim of the present study was to compare the psychological factors for suicidal ideation between adolescents from a psychosocial risk group and from the general population. More specifically, we intend (a) to compare clinical antecedents and history of suicidal behaviors between psychosocial risk adolescents and adolescents in the general population; (b) to evaluate the relationship between risk and protective factors and suicidal ideation in psychosocial risk adolescents; (c) to compare the levels of risk and protective factors for suicidal ideation between the two groups of adolescents; (d) to explore the frequency and impact of negative life events (NLEs) in the two groups; and (e) to assess the direct influence of NLEs, hopelessness, self-esteem, and satisfaction with social support on suicidal ideation in psychosocial risk adolescents and adolescents in the general group.

## 2. Materials and Methods

### 2.1. Participants

A total of 137 adolescents with ages from 10 and 19 years participated (*M* = 14.76; *SD* = 1.40). The total sample was divided in the psychosocial risk group (PRG), composed of 60 participants, and the general group (GG) of 77 participants. The young people in the PRG were recruited under a social project, which exclusively covers young people from neighborhoods coming from families at psychosocial risk.

The participants in the PRG have a mean age of 13.76 years (*SD* = 1.95) and mainly are male (66.7%), and the GG participants have a mean age of 15.53 (*SD* = 0.50) and for the most part are male (53.2%). There were no significant differences between the groups in terms of gender (*ꭓ*^2^ = 2.94, *p* = 0.087, *V* = 0.15), but the participants in the GG were significantly older than those in the PRG (*t* = −6.77, *p* = 0.01, *d* = 1.34).

### 2.2. Instruments

The Personal Data Questionnaire (PDQ), composed of 17 questions, gathers information regarding (a) sociodemographic information; (b) history of psychological disorders, (c) history of self-injurious behaviors and suicidal acts; and (d) history of suicidal acts of family members or close persons.

The Reasons for Living Inventory for Adolescents (RFL-A; [27,28]) assesses the reasons for living considered adaptive and protective, and how these act against suicidal behavior in young people. It consists of 32 items that evaluate five dimensions: family alliance (8 items; e.g., “Feel close to family”; α = 0.89), suicide-related concerns (6 items; e.g., “Thought scares me”; α = 0.85), peer acceptance and support (6 items; e.g., “Friends stand by me”; α = 0.84), future optimism (7 items; e.g., “I like to accomplish”; α = 0.85), and self-acceptance (6 items; e.g., “I accept myself”; α = 0.86). The items have a self-response hypothesis according to a six-point Likert-type scale, from 1 (not at all important) to 6 (extremely important). The total scale of this instrument shows good internal consistency (α = 0.93) and higher scores reveal higher domains’ levels.

The Beck Hopelessness Scale (BHS; [29,30]) assesses the symptoms of hopelessness in relation to future events. It is composed of 20 closed response items (e.g., “My past experiences have prepared me well for my future”; α = 0.64) of a dichotomous format (true/false). Higher values expose higher hopelessness levels.

The Self-Esteem Scale (SES; [31,32]) assesses adolescents’ self-esteem in ten items (e.g., “On the whole, I am satisfied with myself”; α = 0.85) with responses on a 4-point Likert-type scale, from 1 (strongly disagree) to 4 (strongly agree). Higher scores reveal higher levels of self-esteem.

The Negative Life Events Inventory (NLEI; [33]) assesses experienced negative experiences in terms of severity, impact, and frequency. It is composed of 25 items that evaluate four dimensions: adverse family environment (9 items; e.g., “Family conflicts”; α = 0.70), psychological abuse (6 items; e.g., “Humiliations”; α = 0.82), separation and loss (6 items; e.g., “permanent separations (e.g., due to death)”; α = 0.59), and physical and sexual abuse (4 items; e.g., “Severe aggressions”; α = 0.83). It is subdivided into response hypotheses in terms of frequency rated from 0 to 4, from 0 (never) to 4 (very often), and in terms of impact, rated from 1 to 5, with a progressive response from 1 (none) to 5 (extremely negative). The total scale internal consistency is very good (α = 0.88). Higher values expose higher domains’ levels.

The Social Support Satisfaction Scale (SSSS; [34]) is a 15-item instrument that assesses the perception regarding satisfaction with social support through four dimensions: (1) satisfaction with friends (5 items; e.g., “I am satisfied with the amount of friend I have”; α = 0.83); (2) intimacy (4 items; e.g., “Sometimes I feel alone in the world and without support”; α = 0.46); (3) satisfaction with family (3 items; e.g., “I am satisfied with the way I relate with my family”; α = 0.47); and (4) satisfaction with social activities (3 items; e.g., “I miss satisfied social activities”; α = 0.66). Each item is of a self-answer based on a Likert-type scale of agreement from 1 (strongly agree) to 5 (strongly disagree) and higher scores reveal higher dimensions’ levels.

The Suicidal Ideation Questionnaire (SIQ; [35,36]) for the Portuguese population aims to assess the severity of thoughts and cognitions about suicide in adolescents. It is composed of 30 items (e.g., “Thoughts of killing self”; α = 0.95) of a self-answer and relation to the frequency of thoughts/cognitions rated between 0 (I have never had this thought) and 6 (Almost every day). Higher values expose higher suicidal ideation.

### 2.3. Procedure

The data collection was gathered in Algarve (a region in the south of Portugal). The data of young people at psychosocial risk were collected as part of a social project whose goal was to prevent crime, promote social inclusion, and combat the trend towards social exclusion and school absenteeism. As part of this project, the teenagers attended structured activities in classrooms (extra formal curricular activity), so data collection was scheduled with the trainers and carried out while these activities were taking place. The sample of young people from the general population was collected in region secondary schools, with previous authorization from the Directorate General of Innovation and Curriculum Development of the Ministry of Education. The directors of the schools that agreed to take part in the study randomly selected classes whose students met the age criteria and organized a date for the questionnaires to be administered.

In both samples, after the formal authorizations were obtained, a request for authorization was made to the parents and an informed consent was requested from the young people themselves. The collection protocol was applied in groups, in a classroom setting for both samples, always in the presence of a researcher.

### 2.4. Analysis Plan

The data analysis was carried out using the Statistical Package for the Social Sciences (SPSS) statistical data processing program (version 28.0). The Cronbach alpha was computed and scores above 0.70 indicate proper reliability of the factors. Since some of the scales have fewer items, although undesirable, alpha scores between 0.60 and 0.70 can also be accepted. The statistical treatment includes Chi-Square (to compare categorical data) and comparative analyses (to determine significant differences between the mean values of each study group) for each of the variables analyzed, and Student’s t-test for independent samples was performed (after the normality and homogeneity conditions were insured); Pearson’s correlation analysis was performed between negative life events and suicidal ideation, in order to verify the relationship between psychological risk and protective factors and suicidal ideation, along with multiple linear regressions (to understand the direct effects of the variables on suicidal ideation). Effect size was calculated with Cramer’s *V* (varying between 0 and 1) and Cohen’ *d* (0.20 = small effect; 0.50 = medium effect; and 0.80 = large effect). For each of the mentioned analyses, the results are considered significant when the *p* value is less than 0.05 [37].

## 3. Results

In order to meet the objectives outlined, we first conducted a global descriptive analysis of all participants and then separately at the psychosocial risk group (PRG) and the general group (GG) levels. This descriptive analysis refers to the clinical history and suicidal behavior of the self and of family members and friends.

Through the analysis of results obtained in the total sample, it was found that 7 out of 137 participants have already presented a psychological problem (5.1%) and 13 have already received professional psychological help (9.5%). Regarding suicidal behavior, 13 out of 137 participants have already attempted the act with the aim of ending their own lives (9.5%) and 16 have already committed self-injurious acts intentionally (11.7%). In respect to family members and friends, 22 out of 137 had recorded suicide attempts by family members (16.1%) and 37 out of 137 had recorded suicide attempts by friends or close people (27.1%).

We then proceeded to analyze the same indicators in each of the groups (Table 1).

When comparing the two groups, significant differences were found regarding acts with the aim of ending one’s own life (*ꭓ*^2^ = 6.40, *p* = 0.011, Cramer’s *V* = 0.22), which were significantly more prevalent in the PRG.

To explore the relationship between variables—reasons for living, hopelessness, self-esteem, negative life events, and satisfaction with social support—with suicidal ideation, a Pearson’s correlation analysis was performed (Table 2).

In the PRG, there was no significant association between reasons for living (total scale) and suicidal ideation; however, there was a negative association between the subscale of self-acceptance of the RFL-A inventory and suicidal ideation (*r* = −0.31; *p* ≤ 0.05).

In the GG, a negative and statistically significant association was found between reasons for living (total scale) and suicidal ideation, and with all subscales, except the self-acceptance subscale (*r* = −0.22, *p* ≥ 0.05).

Regarding self-esteem, there was a negative and significant association in both groups (PRG: *r* = −0.31, *p* ≤ 0.05; GG: *r* = −0.46, *p* ≤ 0.001), indicating that the more self-esteem an individual has, the lower the propensity for suicidal ideation.

The negative life events and suicidal ideation reveal positive and significant associations, in the PRG and GG groups, with higher scores in total-scale NLE (PRG: *r* = 0.58, *p* ≤ 0.01; GG: *r* = 0.52, *p* ≤ 0.001) and psychological abuse (PRG: *r* = 0.59, *p* ≤ 0.01; GG: *r* = 0.55, *p* ≤ 0.001), and lower correlations in separations and losses (PRG: *r* = 0.32, *p* ≤ 0.05; GG: *r* = 0.37, *p* ≤ 0.001) and physical or sexual abuse (PRG: *r* = 0.33, *p* ≤ 0.01; GG: *r* = 0.23, *p* ≤ 0.05). The adverse family environment subscale shows moderate correlation in the PRG (*r* = 0.57; *p* ≤ 0.01) and low correlation in the GG (*r* = 0.30; *p* ≤ 0.01). The greater the experience of negative life events for an individual, the greater the tendency for thoughts related to suicidal behavior.

In relation to satisfaction with social support, in the PRG, there was a negative correlation between the suicidal ideation and the total-scale SSSS (*r* = −0.39; *p* ≤ 0.01), satisfaction with intimacy (*r* = −0.37; *p* ≤ 0.01), satisfaction with family (*r* = −0.32; *p* ≤ 0.05), and satisfaction with social activities (*r* = −0.29; *p* ≤ 0.05). In the general group, there was also a negative association between satisfaction with social support and suicidal ideation, but with greater intensity in the total scale (total SSSS: *r* = −0.74; *p* ≤ 0.001) and in the subscales (friends: *r* = −0.61, *p* ≤ 0.001; intimacy: *r* = −0.57, *p* ≤ 0.001; family: *r* = −0.53, *p* ≤ 0.001; and social activities: *r* = −0.48, *p* ≤ 0.001). These results suggest that with greater satisfaction with social support, there is less tendency to have suicidal thoughts.

The differences between the psychosocial risk group and the general group were analyzed (Table 3) in relation to the risk factors (i.e., negative life events and hopelessness).

The experience of negative life events (NLEI) showed significant and small effect differences at the total scale (*t*_(102.13)_ = 2.22, *p* = 0.028, *d* = 0.38), a large effect in the category of separations/losses (*t*_(102.56)_ = 2.63; *p* = 0.010; *d* = 0.72), and a small effect in the physical and sexual abuse subscale (*t*_(91.46)_ = 2.22; *p* = 0.027; *d* = 0.39), with higher mean values in the PRG (*M*_NLEI_ = 1.95; *SD*_LEI_ = 1.80; *M*_Separation/Losses_ = 1.97; *SD*_Separation/Losses_ = 2.04; *M*_Physical/Sexual Abuse_ = 1.48; *SD*_Physical/Sexual Abuse_ = 2.42) than in the GG (*M*_NLEI_ = 1.30; *SD*_NLEI_ = 1.28; *M*_Separation/Losses_ = 1.15; *SD*_Separation/Losses_ = 1.45; *M*_Physical/Sexual Abuse_ = 0.68; *SD*_Physical/Sexual Abuse_ = 1.46).

In terms of hopelessness, the results did not reveal statistically significant differences between the groups.

As Table 4 shows, in regard to the comparison of the protective factors between the two groups, there are not significant differences at the level of the total scale of reasons for living (*t*_(112.71)_ = 0.52; *p* = 0.883; *d* = 0.24), nor in the subscales, namely family alliance, concerns about suicide, peer acceptance, optimism for the future, and self-acceptance.

Regarding self-esteem, the results showed highly significant and large effect differences between the two groups (*t*_(134.54)_ = 4.63; *p* ≤ 0.001; *d* = 0.78), showing higher self-esteem in the psychosocial risk group (*M* = 30.22; *SD* = 4.85) than in the general group (*M* = 25.68; *SD* = 6.62).

In satisfaction with social support, significant and large effect differences were found between PRG and GG in relation to satisfaction with family (*t_(_*_126.26)_ = −4.13; *p* ≤ 0.001; *d* = 0.69), and a small effect in the social activities (*t*_(93.39)_ = −2.42; *p* = 0.017; *d* = 0.19), with the psychosocial risk group showing lower values in both (PRG: *M*_Satisfaction Family_ = 9.67, *SD*_Satisfaction Family_ = 1.87; GG: *M*_Satisfaction Family_ = 11.48, *SD*_Satisfaction Family_ = 3.19; PRG: *M*_SocialActivities_ = 9.02, *SD*_SocialActivities_ = 9.72; GG: *M*_SocialActivities_ = 10.35, *SD*_SocialActivities_ = 2.32).

To understand the contribution of the variables to suicidal ideation, we studied the predictors of suicidal ideation, separately, in the RPG (Table 5) and GG (Table 6), using the multiple regression technique. The psychological variables that were significantly correlated with suicidal ideation were included in the model.

The analysis of the results in Table 5 shows that, in the GRP, negative life events, self-esteem, and satisfaction with social support explain 38.0% of the variance in the levels of suicidal ideation, with the contribution of negative life events being positive and quite significant (*p* ≤ 0.001). Although the contribution of self-esteem and satisfaction with social support is negative, it is not significant for explaining the variance of suicidal ideation.

In GG, according to Table 6, these three factors explain 63.0% of the variance of suicidal ideation. All variables have a significant contribution, with the contribution of negative life events being positive (*β* = 0.21, *p* ≤ 0.001) and that of self-esteem (*β* = −0.23, *p* ≤ 0.05) and satisfaction with social support (*β* = −0.56, *p* ≤ 0.01) being negative.

Through a comparative analysis between the two tables presented, it is possible to infer that, in the psychosocial risk group, negative life events seem to be the best predictor of suicidal ideation (*β* = 0.49), while in the general group, all three factors are important, the best predictor being satisfaction with social support. The isolated contribution of negative life events, in this group, is smaller (*β* = 0.21) compared to GRP.

## 4. Discussion

The main objective of this study was to compare the vulnerability to suicidal ideation in young people with and without psychosocial risk; thus, two samples were selected, one as a general group (GG) and the other as a psychosocial risk group (PRG), specifically collected from young people with a psychosocial risk community. The importance of this differentiation between the groups was due to previous studies indicating that the existence of greater psychosocial vulnerability may lead to greater suicidal ideation [38]. In this line of reasoning, young people from families at psychosocial risk are more likely to experience negative events in their environment and in the presence of psychological risk factors, which act as facilitators of suicidal ideation [21,26].

The present study suggests that youth at psychosocial risk committed more acts with the goal of ending their own lives than the general population group. The suicidal process involves social, psychological, and biological variables that can overcome suicidal ideation and go from cognition to the act itself, with the aim of ending one’s own life [39]. Young people with a history of suicide attempts are usually characterized by poor social support, which is consistent with the results obtained, since psychosocially at-risk young people are inserted in contexts with a lack of social resources at various levels [25,38].

In the general population, as expected, we found negative correlations between suicidal ideation and the protective factors (reasons for living, self-esteem, and social support satisfaction) and positive correlations with risk factors (hopelessness and negative life events). Regarding the analysis of the association between risk and protective factors with suicidal ideation in the psychosocial risk sample, negative significant associations were found between suicidal ideation and the self-acceptance of the reasons for living, self-esteem, satisfaction with social support, and also with intimacy and family satisfaction, and positive correlations with negative life events, involving all types of adverse life events.

Self-acceptance is the only subscale of the reasons for living that is negatively associated with suicidal ideation in the PRG. This fact points, on the one hand, to the fact that in the PRG the multiple reasons for living are protective of suicidal ideation and, on the other hand, in the PGR, self-acceptance seems to be the reason more associated with suicidal ideation. These results are congruent with those of self-esteem.

Self-esteem shows a significant and negative association with suicidal ideation, that is, the higher the levels of self-esteem presented by a subject, the lower the tendency to present ideation for suicidal behavior. These data support the idea that self-esteem operates as a protective factor for suicidal ideation in this risk group [23].

A relationship was found between negative life events and suicidal ideation in the psychosocial risk group, that is, the more negative events experienced by the young person, the greater the propensity for suicidal ideation. Adverse events such as an adverse family environment and psychological abuse seem to be more impactful regarding the development of suicidal ideation.

The scientific literature suggests that the effects of social support satisfaction on the development of suicidal behavior indicate that the lower the satisfaction with social support, the greater the vulnerability for adolescents to commit suicide (e.g., [40]). The data obtained in the present study are congruent with the scientific literature, since a significant negative association was found between social support and suicidal ideation, particularly in satisfaction with intimacy in interpersonal relationships and satisfaction with family. To minimize the risk of developing suicidal behavior, it is important to have social support, such as family and friends, who are the most significant relationships for an adolescent. Thus, the less intimacy the adolescent feels with those around them, as well as the less satisfaction they feel with their family, the greater the propensity will be for thoughts related to ending their own life.

The comparative analysis indicates that risk and protective factors may be involved for suicidal ideation. Risk factors are characterized by variables that predispose the development of suicidal ideation [41] such as negative life events. In the present study, the adolescents with psychosocial risk showed higher scores regarding the presence of negative life events, compared to the general group. These data are congruent with the literature, in which youth at psychosocial risk experience many more negative life events than youth not at risk, with more frequency and greater impact [26,41], as well as in the setting of negative life events associated with psychological and sexual abuse, and related to separations and losses, which are experienced more by youth in an adverse social environment than by youth free of psychosocial risk [38,42].

In another sense, there are several protective factors that allow reducing the risk of suicide risk, acting as insulators against it, such as self-esteem [21,43], reasons for living [28], and satisfaction with social support [14,21]. According to the results obtained, at-risk adolescents showed higher self-esteem compared to not-at-risk adolescents, which contradicts the tendency for low self-esteem in this type of population [42]. This may be due to the social skills trained with a social project for over a year, which may have influenced their levels of self-esteem, since one of the project’s objectives is to promote this domain.

Adverse living conditions can influence the family environment of psychosocial risk, as it affects several members of the household, particularly adolescents’ development and well-being, because of a scenario characterized by the lack of family rules and dysfunctional communication [19]. This scenario of family deprivation leads them to a negative perception of the received family support [12]. This study shows agreement in that young people with psychosocial vulnerabilities show less satisfaction with social support than young people without these vulnerabilities. In the perception that young people have of their social support, dissatisfaction with family support stands out quite significantly. Thus, young people have a negative perception of the support provided by their families, and they are also dissatisfied with their participation in social activities, compared to the general group.

According to the associations obtained, there is a need to understand the effect of these variables as possible predictors of suicidal ideation, both in the psychosocial risk group and in the general group. When analyzing possible explanatory models, the model that integrated the variables negative life events, self-esteem, and satisfaction with social support explained 38% of the variance of suicidal ideation in the PRG. The contribution of negative life events proved to be positive and significant, showing greater predictive power than the remaining factors, with negative and non-significant contributions. These three factors proved to be strong predictors in the general group, explaining 63% of the levels of suicidal ideation. Although together they showed a significant contribution, satisfaction with social support had the strongest and most significant contribution.

Based on several studies, negative life events have been found to be a significant predictor for levels of suicidal ideation in youth, with a positive and significant association observed between these two variables (e.g., [44,45]). It has also been found that levels of suicidal ideation are higher the higher the frequency of adverse experiences [46].

It is noteworthy that the results show that negative life events in the psychosocial risk group are the only predictor of the development of suicidal ideation, without requiring the contribution of other variables, while in the general group, the contribution of negative life events is smaller and shared with self-esteem and satisfaction with social support.

Several limitations were found regarding data collection. One refers to participants’ age, since, for intending to study youth from such a specific population (psychosocial risk), a wide range of ages was included (youth between 10 and 19 years old), although it corresponds to the adolescent age group defined by the World Health Organization. Geographic specificity was another limitation, since we only characterize a population of the Algarve region, not allowing results’ generalization. The samples size was also a limitation, since it reflected in, for example, the obtained reliability scores.

The fact that the sample of the psychosocial risk group refers to young people in a social project may represent another limitation of the study, since they were being intervened at the level of prevention of unwellness. This project aimed to prevent crime and promote social inclusion, working on the social skills of adolescents through various promotional activities such as school support, dance and music workshops, and computers, promoting greater well-being. Thus, there may be a regulation of emotions, encouraging young people to perceive and view themselves and the world in a more positive way.

This study also presents relevant contributions, such as highlighting the need for further research on those who are at risk for suicidal ideation, and for having detected the existence of several associated psychological factors. For example, it is known that negative life events are an important risk factor for suicide, and more negative life events are identified in young people from families at psychosocial risk.

Also, it drew attention to the need for an intervention at the level of selective prevention, directed at this specific population, for the promotion of mental health to better ensure the well-being of the young person and promote individual strategies, and for the relationship with others in their environment. Attention was specially drawn to the promotion of positive perceptions and experiences of social support by several significant interveners (e.g., peers, parents, and teachers). Another relevant suggestion refers to the direct intervention with families of the psychosocial group, to empower their abilities to manage their adolescents’ emotions and detect early eventual psychosocial and suicide risks. This type of intervention has the cross-cutting objective of increasing suicide literacy among the different stakeholders, and it is proposed that in addition to the classic face-to-face versions, digital versions could be offered [47].

Considering all the limitations and potentialities mentioned above, we suggest, in terms of research, the repetition of the study with a larger population of Portuguese adolescents and one that is more diverse (i.e., from other regions of Portugal), the extension of the study to other European adolescents using the same instrumentation, and, in terms of practical implications, more targeted programs to specific groups of greater vulnerability, namely, of psychosocial risk, in a suicide prevention direction.

## 5. Conclusions

This study allowed us to identify, analyze, and compare factors associated with suicidal ideation in both samples of young people with and without psychosocial risk. Thus, it was possible to understand the effect that the experience of negative life events has on suicidal ideation in young people with psychosocial risk, compared to young people in the general population. Also, it highlights relevant protection factors that can attenuate potential psychosocial risks.

Several limitations and potentialities were pointed out, reinforcing the importance of programs, at different levels of intervention, that allow for the healthy development of young people. So, it is important to continue the investigation of the predictors of suicide, considering the environment, individual characteristics, and the individual’s role in society, to decrease the prevalence of suicide and associated behaviors, through its prevention and promotion of biopsychosocial well-being.

## Figures and Tables

**Table 1:** Clinical History and Suicidal Behavior in the Psychosocial Risk Group and the General Group.

	PRG (***n*** = 60)	GG (***n*** = 77)	***ꭓ*** ^2^	***p***	***V***
History of psychological problem	4 (67.0%)	3 (3.9%)	0.57	0.451	0.07
Received psychological help	8 (13.3%)	5 (6.5%)	1.84	0.288	0.07
Committed acts with the aim of ending one’s life	10 (16.7%)	3 (3.9%)	6.40	0.011	0.22
Self-injurious acts	10 (16.7%)	6 (7.8%)	2.58	0.109	0.14
Suicide attempts by family members	10 (16.7%)	12 (15.6%)	0.03	0.864	0.02
Suicide attempts by friends or close people	20 (33.0%)	17 (22.1%)	2.36	0.126	0.13

PRG = psychosocial risk group; GG = general group; *ꭓ*^2^ = statistic test; *p* = significance level; *V* = effect size.

**Table 2:** Correlations between RFL-A, BHS, SES, NLEI, and SSSS with SIQ in GRP.

	Suicide Ideation (SIQ)	
	PRG	GG
Reasons for Living (RFL-A)	−0.13	−0.43 ***
Family Alliance	−0.16	−0.28 *
Fear of Suicide	0.09	−0.40 **
Peer Acceptance and Support	−0.18	−0.24 *
Optimism about the Future	−0.03	−0.37 **
Self-Acceptance	−0.31 *	−0.22
Hopelessness (BHS)	0.21	0.61 ***
Self-esteem (SES)	−0.33 **	−0.46 ***
Negative Life Events—Total (NLE)	0.58 **	0.52 ***
Adverse Family Environment	0.57 **	0.30 **
Psychological Abuse	0.59 **	0.55 ***
Separation and Loss	0.32 *	0.37 ***
Physical or Sexual Abuse	0.33 **	0.23 *
Social Support Satisfaction Scale—Total (SSSS)	−0.39 **	−0.74 ***
Satisfaction with Friends/Acquaintances	−0.18	−0.61 ***
Satisfaction with Intimacy	−0.37 **	−0.57 ***
Satisfaction with Family	−0.32 *	−0.53 ***
Satisfaction with Social Activities	−0.29 *	−0.48 ***

* *p* ≤ 0.05; ** *p* ≤ 0.01; *** *p* ≤ 0.001.

**Table 3:** Descriptive statistics and comparison between PRG and GG of the NLEI and BHS.

	PRG (***n*** = 60)		GG (***n*** = 70)		***t***	***p***	***d***
	***M***	***SD***	***M***	***SD***			
Negative Life Events (NLEI)	1.95	1.80	1.30	1.28	2.22	0.028	0.38
Adverse Family Environment	1.78	1.85	1.23	1.30	1.95	0.053	0.34
Psychological Abuse	2.67	2.90	1.15	1.45	1.09	0.274	0.66
Separation and Loss	1.97	2.04	1.15	1.45	2.63	0.010	0.72
Physical and/or Sexual Abuse	1.48	2.42	0.68	1.46	2.22	0.027	0.39
Hopelessness (BHS)	4.04	2.67	3.83	2.75	0.44	0.654	0.08

PRG = Psychosocial Risk Group; GG = General Group; *M* = Mean; *SD* = Standard Deviation; *t* = Statistic Test; *p* = Significance Level; *d* = Effect Size.

**Table 4:** Descriptive statistics and comparison between PRG and GG of the RFL-A, SES, and SSSS.

	PRG (***n*** = 60)		GG (***n*** = 70)		***t***	***p***	***d***
	***M***	***SD***	***M***	***SD***			
Reasons for Living Inventory (RFL-A) Total	4.61	0.83	4.59	0.70	0.14	0.883	0.24
Family Alliance	4.91	0.98	4.84	1.02	0.40	0.690	0.06
Fear of Suicide	3.47	1.57	3.61	1.46	−0.56	0.576	0.09
Peer Acceptance and Support	4.59	1.11	4.77	0.81	−1.08	0.263	0.18
Optimism about the Future	5.00	0.92	4.78	0.71	1.58	0.117	0.26
Self-Acceptance	4.94	1.04	4.85	0.85	0.54	0.588	0.09
Self-Esteem Scale (SES) Total	30.22	4.85	25.68	6.62	4.63	0.000	0.78
Social Support Satisfaction Scale (SSSS) Total	52.55	9.66	54.55	9.01	−1.24	0.215	0.21
Satisfaction with Friends/Acquaintances	20.10	4.92	19.64	4.14	0.60	0.550	0.10
Intimacy	13.76	3.31	13.08	2.27	1.36	0.175	0.20
Satisfaction with Family	9.67	1.87	11.48	3.19	−4.13	0.000	0.69
Satisfaction with Social Activities	9.02	9.72	10.35	2.32	−2.42	0.017	0.19

PRG = Psychosocial Risk Group; GG = General Group; *M* = Mean; *SD* = Standard Deviation; *t* = Statistic Test; *p* = Significance Level; *d* = Effect Size.

**Table 5:** Contribution of the NLEI, SES-Total, and SSSS in explaining suicidal ideation in the Psychosocial Risk Group (*n* = 60).

	Suicide Ideation	
Constant (39.59)		
NLEI—Total	*β* = 0.49 ***	
SES—Total	*β* = −0.10	R^2^ = 0.38
SSSS—Total	*β* = −0.15	

*β* = Standardized Regression Coefficient; *R*^2^ = Coefficient of Determination; NLEI-Total = Total Value of the Negative Life Events Inventory; SES-Total = Total Value of the Self-Esteem Scale; SSSS-Total = Total Value of the Social Support Satisfaction Scale. *** *p* < 0.001.

**Table 6:** Contribution of the NLIE, SES-Total, and SSSS in explaining suicidal ideation in the General Group (*n* = 77).

	Suicide Ideation	
Constant (84.47 ***)		
NLEI—Total	*β* = 0.21 ***	
SES—Total	*β* = −0.23 *	R^2^ = 0.63
SSSS—Total	*β* = −0.56 **	

*β* = Standardized Regression Coefficient; *R*^2^ = Coefficient of Determination; NLEI-Total = Total Value of the Negative Life Events Inventory; SES-Total = Total Value of the Self-Esteem Scale; SSSS-Total = Total Value of the Social Support Satisfaction Scale. * *p* < 0.05; ** *p* < 0.01; *** *p* < 0.001.

## Data Availability

The data presented in this study are available on request from the corresponding author. The data are not publicly available due to licence constraints.
